# Facial cleanliness, environmental factors, and the burden of trachoma in a pastoral conflict area of Tiaty East, Baringo County, Kenya

**DOI:** 10.1371/journal.pgph.0007534

**Published:** 2026-10-27

**Authors:** Loise Wanjira Njogu, George A. Makalliwa, Bridget Kimani, Kellen Karimi, Zephania Kibet, Doris Njomo, Titus Watitu, Paul M. Gichuki

**Affiliations:** 1 School of Public Health, Department of Environmental Health and Disease Control, Jomo Kenyatta Institute of Agriculture and Technology, Nairobi, Kenya; 2 Kenya Medical Research Institute (KEMRI), Graduate School, Nairobi, Kenya; 3 Kenya Medical Research Institute (KEMRI), Eastern & Southern Africa Centre of International Parasitic Control (ESACIPAC), Nairobi, Kenya; 4 Department of Public and Global Health, Faculty of Health Sciences, University of Nairobi, Nairobi, Kenya; 5 Department of Health, Baringo County, Kenya, Government of Baringo; 6 Kenya National Public Health Institute (KNPHI), Ministry of Health, Nairobi, Kenya; ICDDRB: International Centre for Diarrhoeal Disease Research Bangladesh, BANGLADESH

## Abstract

Trachoma remains the leading infectious cause of preventable blindness globally, disproportionately affecting marginalized and impoverished communities. Despite global and national efforts guided by the World Health Organization’s (WHO) SAFE strategy (Surgery, Antibiotics, Facial cleanliness, Environmental improvement), the latter two components remain under-implemented. In Kenya, trachoma remains endemic in 13 of 47 counties, including Baringo. This cross-sectional study was conducted in Tiaty East Sub-County, a trachoma Sub-Evaluation Unit in Baringo, to determine the burden of trachoma and assess facial cleanliness and environmental improvement for control of the disease at the household level. Using a structured questionnaire and observation checklist, data were collected from 178 randomly selected households. A total of 279 children aged 1–9 years were examined for trachomatous inflammation-follicular (TF). The overall TF prevalence was 5.02% (95% CI: 2.8-8.3), with the confidence interval overlapping with the WHO elimination threshold of <5%. Facial cleanliness was sub-optimal, with 91.8% of children having visibly unclean faces. Latrine coverage was low (21.3%), and 99.1% of households without latrines practiced open defecation. Most households (61.8%) sourced water from a dam, and 80.9% required more than 30 minutes to fetch it. Handwashing stations were absent in 94% of households. Final multivariable analysis identified older caregiver age (> 20 years) as significantly associated with lower odds of TF (21–30 years: AOR = 0.089, 95% CI: 0.018–0.447; 31–40 years: AOR = 0.259, 95% CI: 0.068–0.991; ≥ 40 years: AOR = 0.160, 95% CI: 0.029–0.871). Caregiver awareness of poor hygiene as a trachoma-related factor was associated with higher odds of TF (AOR = 3.795; 95% CI:1.272-11.324), suggesting a critical knowledge-practice gap. Although TF prevalence was slightly above the threshold based on a few events, with poor F&E coverage, there remain sub-EU-level gaps in trachoma control. In this context, strengthening the F&E aspects of the SAFE strategy with focused investments on WASH and behavior-based hygiene promotion will be important to the achievement of sub-EU disease elimination.

## Introduction

Trachoma is currently the leading infectious cause of blindness, accounting for approximately 1.9 million cases of blindness globally [1]. Despite the recent efforts to eliminate the disease, trachoma remains a significant public health challenge in most countries [2] with approximately 103 million people globally living in trachoma endemic regions [1]. With 38 countries globally endemic to the disease, they are disproportionately located in Asia, Africa and the Middle East [1]. Sub-Saharan Africa bears a substantial burden of trachoma, as 59% of the countries are endemic, and 85% of the population lives with trachoma [3]. The World Health Organization (WHO) estimates that the annual economic burden attributed to loss of productivity through trachoma is $ 8 billion [1].

The WHO in 1996 embarked on an audacious international program to control and preferably eliminate trachoma under a global program titled “Global Elimination of Trachoma” (GET) by the end of 2020 [4]. One of the targets was to reduce the prevalence of Trachoma Inflammation Folliculitis (TF) to <5% [4]. As part of the program, the WHO implemented the SAFE strategy- a four-pronged plan for trachoma elimination [5]. The comprehensive strategy includes Surgery, Antibiotics, Facial Cleanliness, and Environmental improvement, particularly recommended for trachoma endemic regions [5]. There has been extensive work in the implementation of the SAFE strategy across the globe [4]. Studies have highlighted the efficacy of the strategy, noting significant progress towards the elimination of trachoma. A pilot project rolled out in Kajiado, Kenya, in 2003, found that antibiotics helped reduce TF prevalence from 46.4% to 16.0% [6]. However, the SAFE strategy has been implemented to varying degrees of success [7].There has been a general emphasis on the treatment part, which entails surgery and administration of antibiotics (S&A), with notable inconsistencies in the implementation of the Facial Cleanliness and Environmental improvement (F&E) aspects [8,9].

Facial cleanliness is defined as the absence of nasal and ocular discharges on the face, since these secretions attract eye-seeking flies and facilitate transmission of *Chlamydia trachomatis*, the causative bacterium that causes trachoma [10]. Environmental improvement includes practices that limit fly breeding, including access and proper use of latrines, functional handwashing stations, improved housing conditions (e.g., uncrowded households, adequate separation from domestic animals), and good personal hygiene practices such as not sharing fomites and regularly washing clothes [10,11]. Collectively, these measures reduce both the reservoir of infection (ocular/nasal secretions) and vectors (flies), thereby lowering the probability of transmission [12]. When well implemented, F&E interventions are essential for interrupting transmission and sustaining elimination gains achieved through S&A efforts [13]. However, the effectiveness of F&E implementation depends heavily on water, sanitation and hygiene (WASH) infrastructure and sustained behavioral change in endemic communities, rendering them complex yet indispensable components of trachoma control [10].

In Kenya, trachoma is endemic in 13 counties, including Isiolo, Kajiado, Turkana, Laikipia, Samburu, Marsabit, West Pokot, Embu, Kitui, Narok, Meru, Elgeyo Marakwet and Baringo, with an estimated 2.1 million people living in these areas demanding interventions [14]. The country established the Kenya Trachoma Elimination Program (KTEP), domiciled within the Ministry of Health (MOH), with an ambitious five-year goal to eliminate trachoma as a public health problem by 2019 [15]. Kenya now aims to eliminate the disease by 2027 [16]. In October 2011, the Ministry of Health and the Department of Health Services launched Mass Drug Administration (MDA) for trachoma in the affected counties, with Tiaty East and Tiaty West Sub-Counties of Baringo County being among them [16]. Tiaty East and Tiaty West form part of a trachoma Evaluation Unit(EU) and then had a baseline TF prevalence of 34.3% [16]. Five rounds of MDA were implemented up to 2017, and after an impact survey in 2018, the WHO TF threshold had not been achieved (12.8%), informing three more rounds of MDA between 2020 and 2023 [16]. Unpublished programmatic reports of an impact survey done after the last round of MDA in 2023 showed that the TF prevalence in the EU had dropped to 3.02% [17]. Having achieved the WHO threshold of <5%, the MDA activities were halted [17].

While Baringo County has seen a significant reduction in TF over time, from 34.3% in 2011 [16], to 3.02% in 2023 [17], limited access to the resident population due to insecurity and nomadic lifestyle poses a challenge in effectively reaching members of the community through MDA [16]. Moreso, Tiaty East’s Loyamorok ward has failed to achieve the 80% coverage in MDA across all rounds done between 2012 and 2021 [16]. A similar endemic EU of Kajiado Central has previously reported below target MDA coverage [18]. As of 2024, the EU had a prevalence of 8.0% and therefore has ongoing active surveillance [18]. The consistently low MDA coverage recorded exposes Tiaty East to the possibility of recrudescence of TF [18], making continued disease surveillance in the area imperative. Sub-EU level data will further zoom into the possibility of the need for targeted interventions.

A large proportion of Baringo experiences poor Water, Sanitation, and Hygiene(WASH) infrastructure and is marked as one of the top areas where open defecation is prevalent [19]. In 2022, only 46% of the households had access to sanitation facilities [20]. While F&E enhancement is a more cost-effective and sustainable way of preventing and eliminating trachoma among other infectious diseases, few studies have examined household F&E aspects in marginalized communities. A 2013 formative research that focused on the F&E components of the SAFE strategy in Turkana and Marsabit counties found that F&E components were lagging far behind S&A efforts [21]. This was attributed to poor access to WASH and underlying behavioral practices [21]. While the mentioned formative research shows F&E gaps in a similar setting to Baringo, Turkana has active MDAs ongoing while Marsabit County had TF below the recommended threshold by 2020, while Baringo still had MDA activities ongoing [22].

There is need for evidence on F&E post-MDA cessation that could help identify possible gaps in their adoption and long-term utilization [12]. Poor F&E adoption may lead to the possibility of recrudescence of the disease, which may take time to detect without ongoing active surveillance. With the intersection of consistently below-target MDA coverage, recent MDA cessation and poor WASH infrastructure, Baringo County’s Tiaty East offers an ideal context for our study. In this regard, our study examined the prevalence of TF among children aged from 1 to 9 years in selected households in Tiaty East, Baringo County, and its association with household F&E components of the SAFE strategy.

## Materials and methods

### Ethics statement

Written informed consent translated to the local language (Pokot) was obtained from heads of households or caregivers aged above 18 years for their households to take part in the study, including observation of children for TF. Names of the participants were anonymized by a unique identifier for confidentiality purposes. This study was approved by the Kenya Medical Research Institute (KEMRI) Scientific and Ethics Review Unit (SERU No 4986). A research permit was granted by the National Commission for Science, Technology, and Innovation (NACOSTI) (NACOSTI/P/24/41122). The study further received approval from Baringo County and sub-county offices.

### Study area

The study was conducted in Baringo County (Fig 1), given its historical high TF prevalence, aridity and the population’s nomadic lifestyle [16]. Tiaty East is one of the seven sub-counties of Baringo County that covers an area of 2,106.5 km^2^ with a population of 73,146 and 12,153 households. Tiaty East was specifically chosen for this study as one of the wards in the sub-county consistently reported low MDA coverage (below 80%) [16]. Tiaty East natives are mainly pastoralists who migrate during the dry season and come back to reunite with families during the rainy seasons [16]. The main language of communication in the area is Pokot. Cattle trading, including sheep, goats, cows, camels and donkeys, is their main economic activity. Tiaty East sub-county is also characterized by a wide range of conflicts due to cattle rustling [16]. The map used in Fig 1 was created in ArcGIS® using basemap shapefiles available through the Database of Global Administrative Areas. This data is freely available for use in academic publishing [23]

**Fig 1:**
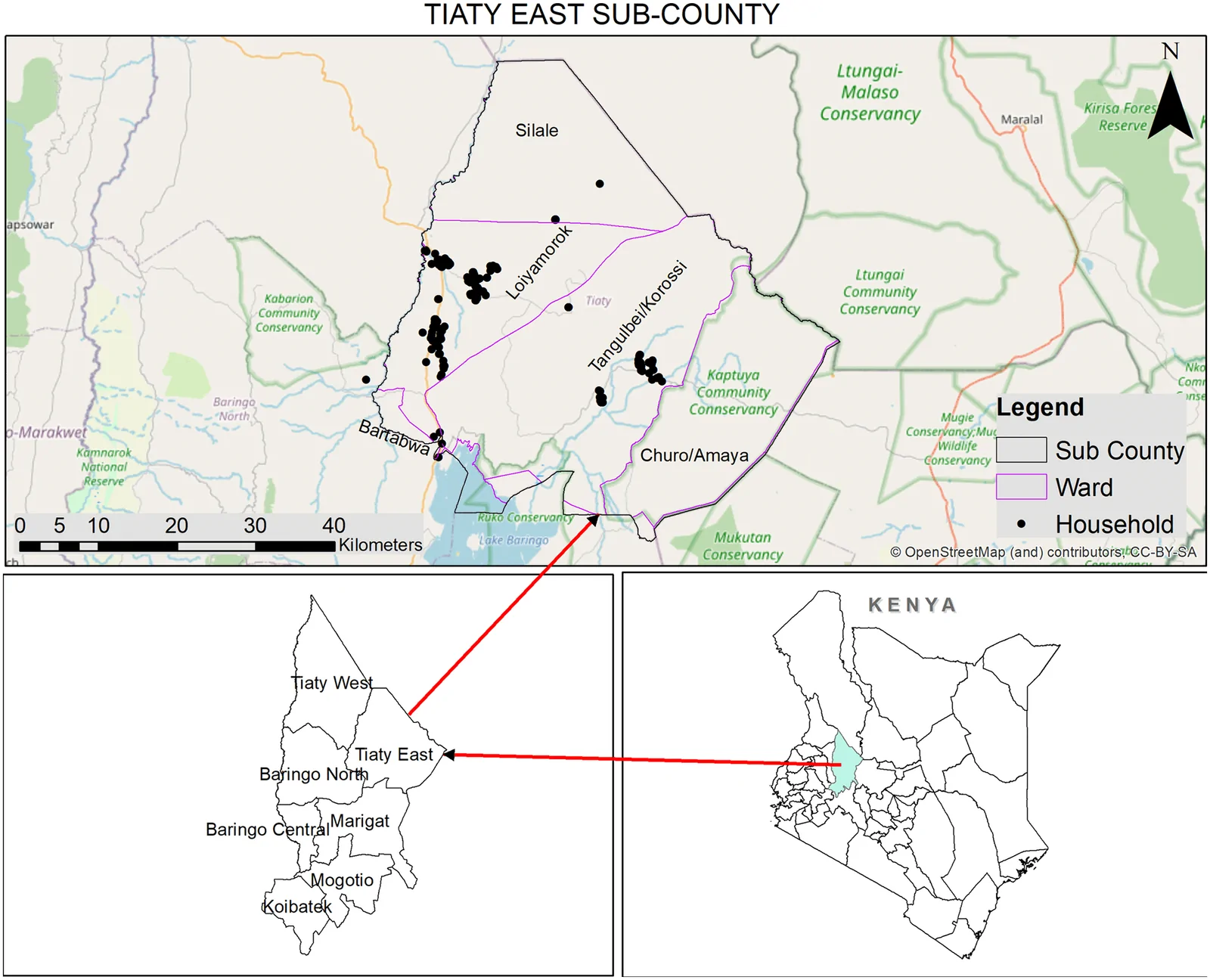
Map of study area showing sampled households. Base layer: Global Administrative Areas (GADM), https://gadm.org/maps/KEN/baringo/tiaty.html. GADM license: freely available for academic use; compatible with CC BY 4.0 for academic publications (https://gadm.org/license.html).

### Study design

This study used a cross-sectional research design, where data on household F&E factors and the prevalence of TF were collected at the same point in time. The identified positive cases were referred for treatment and follow-up by Community Health Promoters (CHPs), who are links between the healthcare systems and the communities.

### Sample size calculation

The sample size was determined using Cochran’s formula with a 95% confidence interval(Z = 1.96) and an error margin(e) of 0.05. We used a prevalence of 5.9%(p = 0.059) in the calculation, obtained from a large population-based study from similar endemic areas in the country [24]. This estimate was chosen as it was considered one that would yield a larger sample size and, consequently, higher statistical precision. A moderate design effect of 2.0 was applied to account for the higher-than-average clustering expected in a nomadic pastoralist setting [25]. This a widely used conservative default [26,27] and also accepted by the WHO for community-based surveys [28]. A 10% non-response rate was assumed, based on similar community-based studies in the region [29]. This led to a sample size of 190 children. At this point, it was assumed that one household would have at least one eligible child, therefore translating to a sample of 190 households.

### Sampling and sample population

The study population comprised households in Tiaty East Sub-county, an area purposively selected due to its endemicity for trachoma and contextual challenges of pastoralism and conflict, which affect the implementation of the SAFE strategy [16]. Within the sub-county, the sampling process followed several levels. A list of all administrative wards was obtained from the Sub- County Disease Surveillance Office. Three wards (Tangulbei, Silale, and Loyamorok) were purposively selected to serve as strata, based on their population distribution and trachoma burden. Within each ward, villages were treated as clusters and randomly selected using simple random sampling. The villages selected were 18 in total: Achukut, Adadat, Atirirai, Chemakil, Chemoril, Cheptaran, Cherelio, Chesimirion, Dapalkow, Lokenoi, Lokrakow, Loruk, Manampelei, Oroo, Pilil, Riongo, Tunatai and Tuwo. The sample size was divided proportionally across the 18 villages using estimated respective village population from the Sub-County offices. Households, the secondary sampling units, were sampled using a random walk method [11]. The random walk sampling was employed by starting from a landmark centrally located in each village until the allocated sample size per cluster was achieved. Households with children between 1 and 9 years old who had lived in the area for at least six months were included in the study (Fig 1).

### Data collection and management

Data was collected between 19^th^ and 26^th^ February 2025. A household pre-tested, structured questionnaire was administered to consenting households by trained research assistants. The F&E exposure variables were established indicators in line with the WHO [30]. Facial cleanliness was observed and operationalized as binary; a child’s face was judged to be ‘clean’ if no discharge from the eyes, nose, dirt, food or flies was observed, and ‘unclean’ if any of these were observed. This was observed at any point between 7am and 6pm while the data was being collected. Environmental improvement variables included latrine use, type of water source, time taken to water source, presence of a hand hygiene facility, housing construction type, household crowding, and proximity to domestic animals. These are standardized indicators provided by WHO [30]

The key outcome variable was the presence or absence of TF in children aged 1–9 years. TF cases were assessed by the research assistants trained by an Ophthalmic Clinical Officer on eye-grading based on a standardized WHO criteria [31], and cases confirmed by certified eye graders. An observational checklist was used to examine hygiene, environmental aspects, and children aged 1–9 years for TF. Confirmed cases were referred to Community Health Promoters (CHPs) for treatment and follow-up. The questionnaire and the observation checklist were administered through the Kobo Collect smartphone application, and data was stored in a centralized server, which was only accessible by the principal investigator.

### Data analysis

The data was cleaned and analyzed using Stata version 17. Responses were assigned numeric identifiers for quantification, analysis and interpretation. The proportion of children with TF and dirty faces (defined as faces with flies, food particles, eye and/or nose secretions), as well as household hygiene practices, was descriptively reported. A multivariable logistic regression model using a Generalized Estimated Equations (GEE) with robust standard errors and exchangeable correlation structure was employed while checking for associations, to account for clustering of children at the village level. Domain-specific models (Demographic characteristics, Housing and living conditions, Hygiene and Sanitation Practices, Knowledge on trachoma disease: cause, transmission and prevention) were fitted to identify variables significantly associated with TF prevalence. Statistically significant variables from these models were then included in a final multivariable model to assess their combined effect. A conceptual framework on how household and caregiver-related predictors are associated with TF outcomes is provided as Fig 2

**Fig 2:**
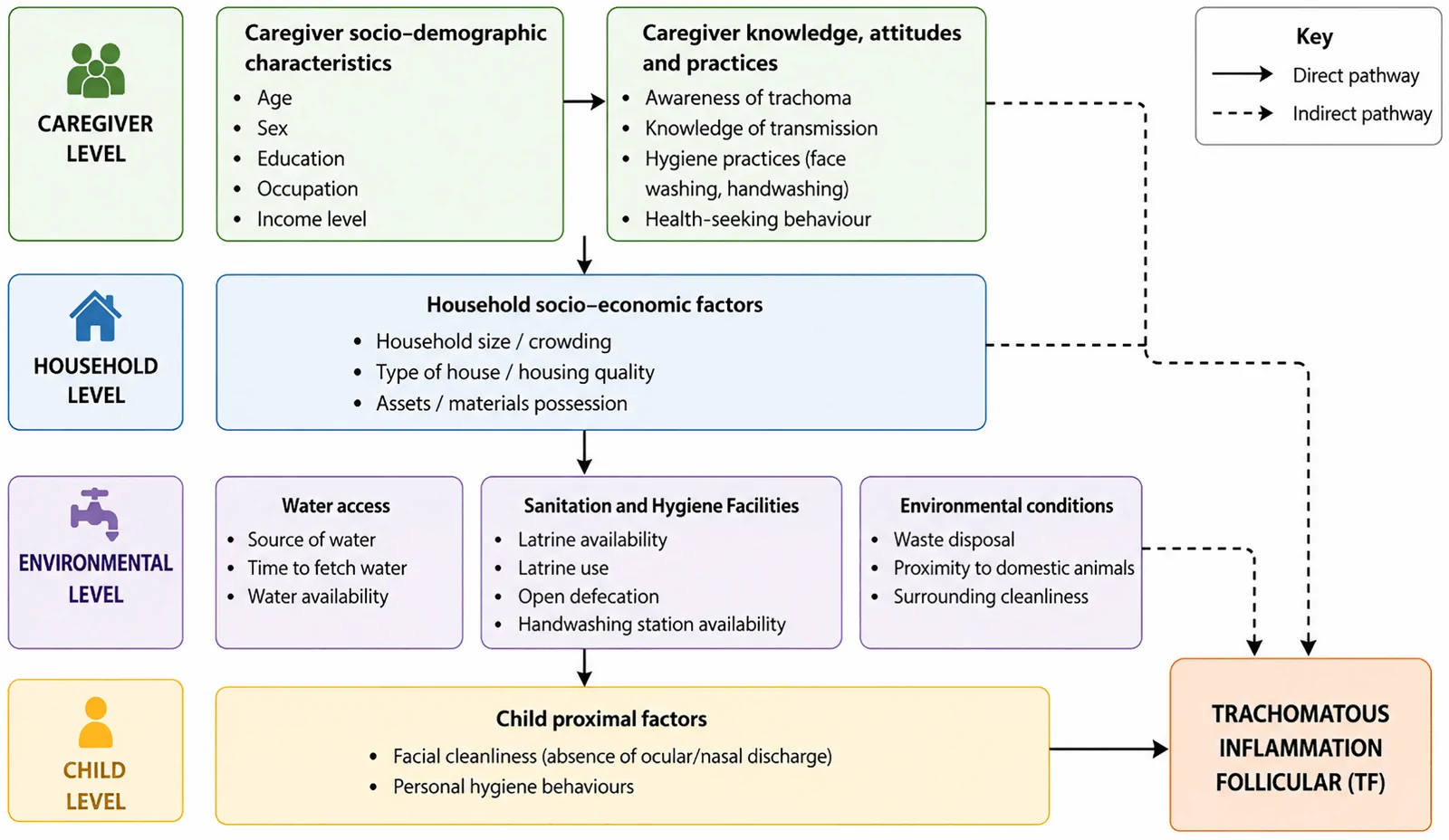
A conceptual framework on factors associated with TF among children aged 1-9 years.

## Results

### Response rate

At data collection, we were able to achieve a sample of 178 households in Tiaty East Sub-County. Within these 178 households, 279 children were observed for TF and facial cleanliness. This is a 94% response rate. A response rate of at least 85% in cross-sectional studies is considered sufficient to achieve target parameters [32].

### Socio-demographic and economic characteristics

Of the 178 households sampled, most primary caregivers were female (80.3%) and aged 21–30 years (45.5%). Over half (53.9%) had no formal education, and 43.8% earned a monthly household income of only $7.76–38.75, reflecting the economic vulnerability of the study population. Household crowding was noted, with a mean of 5.93 members (SD ± 2.47) and 60.1% of households having only one sleeping room. The majority of households (91.0%) had no means of transport, highlighting limited household assets in this population. Detailed socio-demographic and socio-economic characteristics are presented in Tables 1 and 2.

**Table 1:** Socio-demographic characteristics.

Variable	n	(%)
**Age of Primary Caregiver**		
≤20	7	3.9
21 - 30	81	45.5
31 - 40	48	27.0
≥41	42	23.6
**Sex of Caregiver**		
Female	143	80.3
Male	35	19.7
**Religion**		
Christian	133	74.7
Muslim	1	0.6
Pagan	44	24.7
**Marital Status**		
Divorced/Separated	9	5.1
Married	143	80.3
Unmarried/Single	9	5.1
Widowed	17	9.5

**Table 2:** Socio-economic characteristics.

Variable	n	(%)
**Caregiver education level**		
Some form of education	82	46.1
No Education	96	53.9
**Employment status of household head**		
Employed/Business	146	82.0
Unemployed	32	18.0
Monthly household Income ($*)		
≤7.75	19	10.7
7,76 - 38.75	78	43.8
38.76 – 77.51	55	30.9
≥77.52	26	14.6
**Type of House**		
Iron Sheets	31	17.4
Mud	35	19.7
Grass/Thatched	56	31.5
Stone	27	15.1
Timber	29	16.3
**Number of rooms for sleeping**		
1	107	60.1
2	48	27.0
3	18	10.1
4	3	1.7
5	2	1.1
**Number of household members**		
Mean: 5.93 (SD ± 2.47), Min 2, Max 17		
**Means of transport**		
None	162	91.0
Motorcycle	15	8.4
Car	1	0.6

*Converted from Kenyan Shillings (KES) using the rate 1 KES = $129.

### Prevalence of trachoma inflammation folliculitis

The study found that 5.02% (95% CI: 2.8–8.3%) of 279 children observed in the households had TF. This was slightly higher than the 3.02%(95% CI: 2.2–4.0%) reported prevalence in 2023 EU level impact survey [17]. The difference between the two was, however, not statistically significant (p = 0.084). These results have been presented in Table 3

**Table 3:** TF prevalence.

Trachoma	n (%)	95% CI	EU81962	95% CI	p-value
**No**	265(94.98)	91.7 –97.2	1607(96.98)	96.0 – 97.8	0.084
**Yes**	14(5.02)	2.8 – 8.3	50(3.02)	2.2 – 4.0	
**Total**	279(100)		1657(100)		

### Facial cleanliness

Facial uncleanliness was observed with 91.8% of children having visibly unclean faces with either dirt on their face, nasal and eye discharge, pointing at poor implementation of the Facial cleanliness component of the SAFE strategy (Table 4)

**Table 4:** Facial cleanliness and environmental factors.

Variable	n (%)
**Facial Cleanliness**	
**Any Dirt on the Face**	
No	188 (67.4)
Yes	91 (32.6)
**Discharge from the Eye**	
No	192 (68.8)
Yes	87 (31.2)
**Discharge from the Nose**	
No	204 (73.1)
Yes	75 (26.9)
**Flies on the Face**	
No	242 (86.7)
Yes	37 (13.3)
**Overall Facial Cleanliness**	
Not Clean	256 (91.8)
Clean Face	23 (8.2)
**Environmental Improvement Factors**	
***Household hygiene and sanitation facilities***	
**Working Latrine in Compound**	
No	140 (78.7)
Yes	38 (21.3)
**Handwashing Station**	
No	168 (94.4)
Yes	10 (5.6)
**Fly Traps**	
No	161 (90.5)
Yes	17 (9.5)
**Household’s Primary Source of Water**	
Dam	110 (61.8)
Piped Water	13 (7.3)
Stream/River	27 (15.2)
Borehole	28 (15.2)
**Time Taken to Water Source (One Way)**	
Water source in the compound	5 (2.8)
Less than 30 minutes	29 (16.3)
30 minutes to 1 hour	136 (76.4)
More than 1 hour	8 (4.5)
**Water Storage**	
Barrels > 20 liters	46 (25.8)
Containers ≤ 20 liters	123 (69.1)
Tanks > 1000 liters	9 (5.1)
**Closed Water Storage**	
Yes	121 (68.0)
No	57 (32.0)
***Household hygiene and Sanitation practices***	
**Face Washing Frequency**	
Once a day	56 (31.5)
Two times a day	55 (30.9)
Three times a day	42 (23.6)
Over three times a day	25 (14.0)
**Face Washing Means**	
Water Only	92 (51.7)
Soap and Water	86 (48.3)
**Clothes Washing Frequency**	
More than once a week	93 (52.2)
Once a week	70 (39.3)
Two times a month	6 (3.4)
Once a month	9 (5.1)
**All Members Using Latrine**	
Yes	36 (94.7)
No	2 (5.3)
**Reason for Not Using Latrine**	
Cultural reasons	82 (57.8)
Preference	58 (40.8)
Other Reasons	2 (1.4)
**Latrine Cleaning**	
Daily	19 (50.0)
Once a week	10 (26.3)
More than a week	4 (10.5)
Never	5 (13.6)
**If Latrine Absent/Not Used, Where They Defecate**	
Bush/Compound	140 (99.1)
Shared latrines	2 (0.9)
**Livestock Present**	
Yes	141 (79.2)
No	37 (20.8)
**Where Animals are Kept**	
In the house	21 (14.9)
20m or less from the house	49 (48.9)
>20m from the house	51 (36.2)
**Visible Feces on Compound**	
No	57 (32.0)
Yes	121 (68.0)
**Garbage in the Compound**	
No	146 (82.0)
Yes	32 (18.0)

### Environmental improvement

#### Hygiene and sanitation facilities.

Latrine coverage was low with 78.7% lacking a working latrine in their compound, and 94.4% lacking a handwashing station. This shows a gap in some of the key aspects of hygiene and sanitation. Other aspects, including household water source, time taken to water source and water storage, are shown in Table 4.

#### Hygiene and sanitation practices.

Open defecation was common, with 99.1% of households without a functional latrine or with non-compliant members defecating in a bush or open compound, driven predominantly by cultural reasons (57.8%) and personal preference (40.8%). Livestock were present in 79.2% of households, with 48.9% kept within 20 meters of the house, and visible feces were observed in 68.0% of compounds. These are conditions that facilitate fly breeding and trachoma transmission. Full hygiene and sanitation practice data are presented in Table 4.

### Factors associated with the prevalence of Trachoma Folliculitis

Prior to multivariable modelling, multicollinearity was assessed using the variance inflation factor (VIF). The mean VIF was 3.96 with all individual values below 10, indicating no evidence of severe multicollinearity (Table A in S1 Appendix). Model discriminatory ability was good (AUC = 0.87, 95% CI: 0.77–0.97), exceeding the accepted threshold for adequate discrimination (Table B in S1 Appendix). Sensitivity analyses across four model specifications confirmed consistency in the direction and magnitude of all odds ratios (Table C in S1 Appendix). Given the low TF prevalence, associations reported should be interpreted as exploratory and hypothesis-generating rather than definitive.

Parents or caregivers above 20 years were significantly less likely to have a child with TF as compared to younger caregivers 20 years or less. However, the likelihood varied with age, with 21–30 years (AOR = 0.027, 95% CI: 0.002–0.306, *p* = 0.004), 31–40 years (AOR = 0.049, 95% CI: 0.004–0.586, *p* = 0.017), and ≥40 years (AOR = 0.028, 95% CI: 0.002–0.471, *p* = 0.013). Additionally, households with a monthly income of $38.76-77.51 had significantly lower odds of having a child with TF (AOR = 0.058, 95% CI: 0.004–0.851, *p* = 0.038), compared to the lowest income group. Male caregiver sex, marital status, education level, and religion were not significantly associated with TF prevalence (Table 5).

**Table 5:** Association of demographic characteristics and TF prevalence.

Variable	Crude Odds Ratio	P-value	95% CI	Adjusted Odds Ratio	P-value	95% CI
**Caregiver’s Age**						
≤20	REF			REF		
≥21 - 30	0.042	<0.001	[0.008, 0.218]	0.027	**0.004**	[0.002, 0.306]
≥31 - 40	0.112	0.004	[0.025, 0.503]	0.049	**0.017**	[0.004, 0.586]
≥40	0.056	0.001	[0.009, 0.331]	0.028	**0.013**	[0.002, 0.471]
**Caregiver’s Sex**						
Female	REF			REF		
Male	3.666	0.024	[1.182, 11.370]	3.175	0.169	[0.613, 16.461]
**Caregiver’s Marital Status**						
Divorced	REF			REF		
Married	0.366	0.346	[0.045, 2.968]	0.307	0.341	[0.027, 3.488]
Unmarried/Single	1.751	0.643	[0.164, 18.657]	0.157	0.353	[0.003, 7.814]
Widowed	0.361	0.475	[0.022, 5.893]	0.700	0.844	[0.020, 24.543]
**Caregiver’s Education**						
No education	REF			REF		
Some level of education	0.951	0.929	[0.311, 2.909]	0.892	0.898	[0.155, 5.124]
**Household Monthly Income ($)**						
≤7.75	REF			REF		
≥7.76 - 38.75	0.458	0.264	[0.116, 1.804]	0.277	0.185	[0.042, 1.846]
≥38.76 - 77.51	0.079	0.043	[0.007, 0.921]	0.058	**0.038**	[0.004, 0.851]
≥77.52	0.184	0.150	[0.018, 1.838]	0.353	0.445	[0.024, 5.107]
**Religion**						
Christian	REF			REF		
None	1.941	0.247	[0.631, 5.970]	4.125	0.122	[0.684, 24.884]

REF = Reference category.

After adjusting for confounding factors, none of the variables within the domain of Housing and Living Conditions was significantly associated with trachoma prevalence (Table 6).

**Table 6:** Association of housing and living conditions and TF prevalence.

Variable	Crude Odds Ratio	P-value	95% CI	Adjusted Odds Ratio	P-value	95% CI
**Type of House**						
Other (specify)	REF			REF		
Timber/Iron sheet/Stone	0.494	0.254	[0.147, 1.663]	0.713	0.661	[0.157, 3.235]
**Number of Rooms used for sleeping**						
1 room	REF			REF		
2 rooms	0.227	0.159	[0.029, 1.782]	0.264	0.250	[0.027, 2.561]
3 rooms	0.774	0.742	[0.168, 3.571]	0.409	0.443	[0.042, 4.014]
**Number of Household Members**						
Less than 4	REF			REF		
More than 4	1.478	0.612	[0.326, 6.704]	1.374	0.701	[0.271, 6.963]
**Livestock kept**						
No	REF			***		
Yes	2.57	0.362	[0.337, 19.584]			
**Where livestock are kept**						
In the house	REF			REF		
Outside the house	0.659	0.517	[0.187, 2.327]	0.602	0.424	[0.173, 2.093]

***Omitted due to collinearity.

After adjustment, no hygiene or sanitation variable was significantly associated with TF prevalence. Latrine use (AOR = 0.271, 95% CI: 0.062–1.183, p = 0.082), face washing more than once daily (AOR = 0.790, 95% CI: 0.258–2.414, p = 0.679), and the presence of fly traps (AOR = 2.267, 95% CI: 0.244–21.048, p = 0.472) all showed non-significant associations. These null findings likely reflect the near-universal absence of sanitation and hygiene infrastructure across households, which limited the statistical contrast needed to detect associations. Full results are presented in Table 7.

**Table 7:** Association of hygiene and sanitation practices and TF prevalence.

Variable	Crude Odds Ratio	P-value	95% CI	Adjusted Odds Ratio	P-value	95% CI
**Face washing frequency**						
Once a day	REF			REF		
More than once	0.473	0.175	[0.160, 1.396]	0.790	0.679	[0.258, 2.414]
**Face washing means**						
Soap and Water	REF			REF		
Water only	0.977	0.967	[0.320, 2.986]		0.592	[0.223, 2.447]
**Clothes washing frequency**						
More than once a day	REF			***		
Once a day	2.114	0.175	[0.716, 6.240]			
**Latrine use by household members**						
Cultural reasons	REF			REF		
Preference	0.260	0.058	[0.064, 1.049]	0.271	0.082	[0.062, 1.182]
**Existence of fly traps**						
No	REF			REF		
Yes	0.846	0.870	[0.115, 6.251]	2.267	0.472	[0.244, 21.048]

***Omitted due to collinearity.

Caregiver knowledge of the cause of trachoma was significantly associated with TF. Compared to caregivers who attributed trachoma to witchcraft or curses, those who identified flies (OR = 0.14, 95% CI: 0.023–0.860, p = 0.034) or eye infection (OR = 0.047, 95% CI: 0.003–0.725, p = 0.028) as the cause had significantly lower odds of having a child with TF (Table 8). Regarding transmission, caregivers who identified poor personal hygiene as a transmission route had significantly higher odds of TF (AOR = 8.592, 95% CI: 1.133–65.169, p = 0.037), while caregivers who identified a dirty environment as a transmission factor had lower odds (AOR = 0.283, 95% CI: 0.083–0.965, p = 0.044). Knowledge of other transmission routes, including contact with infected discharge, sharing linen, and fly exposure, was not significantly associated with TF. Full results are presented in Table 9.

**Table 8:** Knowledge on causes of trachoma and TF prevalence.

Variable	Odds Ratio	P-value	95% CI
**Cause of Trachoma**			
Witchcraft/Curse	REF		
Flies	0.14	**0.034**	0.023 - 0.860
Infection of the Eye	0.047	**0.028**	0.003 - 0.725

**Table 9:** Association of knowledge about trachoma transmission and TF prevalence.

Variable	Crude Odds Ratio	P-value	95% CI	Adjusted Odds Ratio	P-value	95% CI
**Contact with discharge of infected people**						
No	REF					
Yes	0.508	0.269	[0.152, 1.690]	0.459	0.173	[0.150, 1.407]
**Flies**						
No	REF					
Yes	1.379	0.693	[0.280, 6.791]	1.445	0.646	[0.301, 6.932]
**Sharing linen/clothes**						
No	REF					
Yes	1.017	0.978	[0.315, 3.281]	0.965	0.953	[0.299, 3.115]
**Poor personal hygiene**						
No	REF					
Yes	4.744	0.140	[0.601, 37.434]	8.592	0.037 **	[1.133, 65.169]
**Dirty environment**						
Yes	REF					
No	2.804	0.101	[0.817, 9.623]	0.283	0.044 **	[0.083, 0.965]

Before adjustment, lack of knowledge on trachoma prevention was significantly associated with TF (Crude OR = 9.906, 95% CI: 1.675–8.576, p = 0.01). There was, however, no significant association between the caregiver knowledge of trachoma prevention and the presence of TF after adjustment (Table 10).

**Table 10:** Association of knowledge on trachoma prevention and TF prevalence.

Variable	Crude Odds Ratio	P-value	95% CI	Adjusted Odds Ratio	P-value	95% CI
**Avoid overcrowding**						
No	REF			REF		
Yes	0.567	0.588	0.073 – 4.424	0.568	0.368	0.165 – 1.948
**Not Knowing**				***		
No	REF					
Yes	9.906	**0.011**	1.675 – 8.576			
**Keeping the environment clean**						
No	REF			REF		
Yes	1.445	0.653	0.291 – 7.175	1.467	0.682	0.234 – 9.177
**Not living with animals**						
No	REF			REF		
Yes	0.459	0.230	0.129 – 1.637	0.466	0.218	0.138 – 1.571
**Not sharing linen**						
No	REF			REF		
Yes	0.243	0.074	0.051 – 1.145	0.243	0.099	0.045 – 1.304
**Proper Disposal of Waste**						
No	REF			REF		
Yes	0.763	0.663	0.226 – 2.573	0.89	0.834	0.300 – 2.639
**Washing Face and Hands**				***		
No	REF					
Yes	0.718	0.639	0.180 – 2.862			

***Omitted due to collinearity.

A combined domain-wise multivariate GEE model revealed that the age of the caregiver was significantly associated with the presence of TF, with older caregivers above 20 years being less likely to have a child with trachoma. Knowledge of poor personal hygiene as an associated factor was also significantly associated with TF prevalence (AOR = 3.795, p = 0.017). Time taken to fetch water and knowledge of the causes of trachoma were not significantly associated with the disease (Table 11).

**Table 11:** Final model of factors associated with TF prevalence.

Variable	Adjusted Odds Ratio	P-value	95% CI
**Age of primary caregiver**			
≤20 years	REF		
21–30 years	0.089	**0.003**	0.018 – 0.447
31–40 years	0.259	**0.048**	0.068 – 0.991
≥40 years	0.160	**0.034**	0.029 – 0.871
**Income ($)**			
≤7.75	REF		
≥7.76 - 38.75	0.618	0.515	0.145 – 2.633
≥38.76 - 77.51		****	
≥7.52	1.171	0.901	0.097 – 14.128
**Time to fetch water**			
1 hour	REF		
30 minutes to 1 hour	0.061	0.059	0.003 – 1.114
**Poor Personal Hygiene as an associated factor**			
No	REF		
Yes	3.795	**0.017**	1.272 – 11.324
**Dirty Environment**			
No	REF		
Yes	0.583	0.277	0.221 – 1.541
**Knowledge about the cause of Trachoma**			
Witchcraft/Curse	REF		
Flies	0.156	0.051	0.024 – 1.007
Infection of the eye/Bacterial infection	0.117	0.181	0.005 – 2.707

****Predicts failure perfectly.

## Discussion

### TF prevalence

This study established a trachoma prevalence of 5.02% (95% CI: 2.8-8.3) in Tiaty East (a sub-EU). This is close to the WHO trachoma elimination target of <5.00% [4],and the confidence intervals overlap with the cut-off, introducing significant uncertainty about the true sub-EU TF burden. A post-MDA impact survey done in 2023 in Tiaty East and Tiaty West sub-counties reported a prevalence of 3.02% (95% CI: 2.2 – 4.0) [17], which is below the WHO threshold for elimination. This impact survey showed a decrease from 12.80% in 2018 [33], suggesting overall programmatic progress. However, the sub-EU prevalence reported in our study may indicate heterogeneity within the EU, with possible “pockets” of active trachoma infection in Tiaty East. Since the EU impact survey had a larger sample size and study area, our study points to the possible need for continued surveillance and targeted treatment the sub-EU level.

### Facial cleanliness

The current study shows facial cleanliness is a challenge in the study area, with 91.8% of children observed having dirty faces. Although direct comparisons across settings should be interpreted carefully due to differences in measurement approaches and context, this proportion appears higher than reports from different contexts. In a study conducted in Senegal, where the prevalence was within the WHO targets, 62.6% of the children had dirty faces [34]. A study in Ethiopia, which carries 62% of the global trachoma burden [35], found that 78.2% of the children had dirty faces [8], which is lower than the current study. Our findings concur with the aforementioned studies [8,36] that facial uncleanliness is a persistent issue in some of the most affected African countries. Facial cleanliness remains a critical determinant for trachoma, as children with ocular or nasal discharge on their faces are significantly more likely to harbor infection [11]. This study highlights persistent gaps in facial cleanliness among children in Tiaty East and emphasizes the urgent need for strengthened behavior change communication.

### Environmental improvement

This study has established that gaps in several environmental improvement infrastructures and practices for trachoma control exist. On latrine infrastructure and use, our study found that 78.7% of households surveyed lacked latrines, with 99.1% of these practicing open defecation. These findings are consistent with an impact evaluation survey conducted after the last MDA in the region, which reported 82.9% of households without latrines and 82.2% practicing open defecation [17]. In contrast, studies from Ethiopia show much higher latrine coverage despite a higher TF prevalence of 21.16% [2]. Another study in the area also found that only 2.3% of households lacked latrines and 0.5% of household members practiced open defecation [37]. Such stark differences in latrine ownership and use may be explained by cultural norms and economic contexts. For example, a study in Kenya observed a reluctance among some households to share latrines, even within families [38], highlighting that access alone does not guarantee use. While open defecation sustains fly breeding and facilitates continued transmission of trachoma within communities, this study design does not allow causal inference between sanitation practices and TF at the individual level. Nonetheless, these factors may pose a challenge in breaking trachoma transmission patterns in the area.

Keeping livestock close to dwellings increases exposure to animal feces, which serve as breeding sites for flies [39]. While several studies have shown that domestic animal pens located very close to human dwellings are associated with a higher odds of TF [40–42], 63.8% of our sampled households fell in this category. In addition, 68.0% of compounds had visible feces, reflecting gaps in environmental hygiene similar to those reported in pastoralist communities in Ethiopia [43]. Studies from settings with improved sanitation and waste management have documented lower fly densities and reduced trachoma transmission [11]. Encouragingly, 82.0% of households reported no garbage within the compound, a positive finding considering that improper solid-waste disposal has been shown to significantly increase trachoma prevalence from studies done in Ethiopia [44] and other African settings [45]. These results highlight both progress and persistent gaps in the environmental improvement component of the SAFE strategy. However, in this cross-sectional analysis, these factors could not be statistically linked to TF, limiting conclusions about their independent contribution.

Water accessibility is a critical determinant for the effectiveness of the F&E components of the SAFE strategy for trachoma elimination. Beyond availability, water sources must be safe for household use, as emphasized by the WHO [46,47]. In the present study, only 22.5% of households reported access to safe water sources (piped water and boreholes), and just 2.8% had these sources located within their compound. Furthermore, for both safe and unsafe water sources, 80.9% of households reported a one-way collection time of 30 minutes or more. These findings highlight a significant gap in accessibility to the very resource necessary for sustaining hygiene and sanitation in a household. Improved access to safe water, within or close to households, not only supports better facial cleanliness and environmental hygiene but also facilitates the broader social and behavioral changes required to interrupt trachoma transmission and reduce the burden of other infectious diseases [8,48].

The presence of hand washing stations in the household or compound is recognized as a key environmental improvement factor for trachoma control, since contaminated hands are a known route of trachoma transmission [49]. In our study, 94.4% did not have a hand-washing facility. While studies have shown facial cleanliness can also be promoted by the presence of handwashing stations, which can also be used as face washing stations [48], the prevalence of handwashing stations in many trachoma‐endemic regions is very low. In Amhara, Ethiopia, only 13.9% of households had a hand washing station [50], while a more recent study reported 26.0% having a handwashing facility [51]. These consistent findings show a need for more advocacy for hand washing stations that may, in turn, improve both facial and environmental hygiene aspects for trachoma control.

### Associated factors

Our study suggests that children with caregivers above the age of 20 years have lower odds of infection. While several studies have investigated the factors associated with TF in a similar setting [52–54], few studies noted caregiver age as a possible independently associated factor. This suggests a possibility of the predictor being neglected in analysis, or not strongly associated as seen in a study in Ethiopia [54].

Households earning more were less likely to have a child with TF, a finding that aligns with wider evidence, which indicates that trachoma is highly associated with poverty [55]. In addition, while previous studies found that the size of the family was associated with high levels of transmission [42,56], this study did not find any association between family size and prevalence of TF. These differences may reflect limited ability to detect associations, potentially due to the low prevalence of TF in the study population. The findings in this study also contradict the findings of a study in Kenya [33] and Tanzania, [57], that found a significant negative association between being a Christian and a Muslim and trachoma, respectively. This necessitates further research on the topic to establish how various aspects of religion may relate to the prevalence of TF.

While our descriptive statistics show that the study area has major gaps in hygiene and sanitation aspects for trachoma control, we did not find these factors significantly associated with TF. These findings contradict evidence from previous studies, which suggest that poor sanitation and hygiene contribute significantly to the spread of trachoma [58]. The study also contradicts the findings from a study in Ethiopia [45]. The systematic literature review and meta-analysis established that absence of latrine, unclean faces and unutilized soap for washing was significantly associated with TF among children [45]. However, the significant association of caregivers’ knowledge of poor personal hygiene as a related factor for trachoma emphasizes the findings of several studies that knowledge does not always translate to practice [38,59–61]. There is therefore a need to focus on behavioral change in the area and similar endemic settings to break trachoma transmission.

## Conclusion and recommendations

The cross-sectional data on TF prevalence presented in this study showed that the level of TF prevalence among children aged 1–9 years was 5.02% (95% CI: 2.8 – 8.3%) in Tiaty East Sub-County, which was slightly higher than the < 5% elimination target set by WHO. However, the confidence interval crosses the elimination threshold, making it impossible to draw a definitive conclusion from our data on the Sub-EU elimination status. A larger survey is needed before making definitive conclusions.

At the household level, there was close to a universal lack of F&E infrastructure and practice, with most children having unclean faces, most households without latrines and handwashing facilities and the majority using remote and unprotected water sources. In the adjusted model, older age of the caregiver was associated with reduced odds of TF, and awareness of poor hygiene as an associated factor for trachoma was associated with increased odds of disease, highlighting an important knowledge-practice gap. These findings collectively suggest that MDA alone is not enough to drive the elimination of active trachoma in this context and that a need exists for targeted and context-specific investment in F&E.

As a unique sub-EU-level investigation of household F&E determinants of TF in a post-MDA pastoralist setting in Kenya, this study offers an exploratory evidence base for targeted program planning towards trachoma elimination. We recommend that the Baringo County Government and WASH partners direct efforts towards improving latrine coverage, handwashing facility provision, and water access in Tiaty East. Hygiene promotion must move beyond awareness-based messaging towards practical, behavior-change approaches, with particular focus on young caregivers. Additionally, TF prevalence and F&E indicators should be incorporated into the primary healthcare reporting system through existing Community Health Promoters, enabling a passive sub-county surveillance to detect early signs of trachoma recrudescence.

## Limitations of the study

This study highlights important aspects of trachoma control but is subject to certain limitations. The nature of our study adopting a cross-sectional study design, makes it exploratory and therefore direct or indirect causation cannot be established. The data was collected at the sub-EU level, limiting direct comparability with EU-level data. Conducting a similar study in Tiaty West would provide a stronger basis for comparison and help identify which areas carry the greatest burden related to gaps in F&E implementation. Further to this, our reported prevalence is near the elimination threshold. A larger study within the Sub-EU will achieve a more precise estimate.

The use of a random walk method for household selection may have introduced selection bias, as households located closer to main paths or more accessible areas may have had a higher probability of being included. Since household selection was conditional on the presence of at least one child between the ages of 1–9, sampling probability was not necessarily equal for all households but was implicitly a function of household composition. The probability of inclusion might have been slightly greater for households that had children in the target age group, or for those in neighborhoods with more children. This should be considered when interpreting the generalizability of our findings.

The questionnaire relied partly on self-reported information, which may be subject to respondent bias. However, this was mitigated using an observation checklist to validate key responses. Future studies should aim to address these limitations by incorporating broader geographic coverage and triangulating data sources.

The small number of TF cases limited the number of covariates that could be included in multivariable models, increasing the possibility of residual confounding and making the findings exploratory. Clustering at village level was accounted for using GEE. However, residual within-household correlation among children in the same household may remain. All these should be considered while interpreting our study.

## Supporting information

S1 Appendix Supplementary tables. (DOCX)
