# Supplementary material for: Facial cleanliness, environmental factors, and the burden of trachoma in a pastoral conflict area of Tiaty East, Baringo County, Kenya
Source: PLOS Glob Public Health. 2026 Oct 27;6(10):e0007534. doi: 10.1371/journal.pgph.0007534 (PMC13712619; doi:10.1371/journal.pgph.0007534)
Supplement: Supplementary file 1 [file pgph.0007534.s003.pdf]

Manuscript title: Facial cleanliness, environmental factors and the burden of trachoma in a pastoral conflict area of Tiaty East Baringo County, Kenya

Manuscript Number PGPH-D-25-03058

Comments reviewer:

Abstract

The abstract highlights the global burden of Trachoma, emphasizing the status of the leading infectious cause of preventable blindness. However, the objective is not explicitly written. It seems unclear for the reader.

In the results part, the author more likely focused on descriptive analysis, while regression analysis are poorly articulated; without these, the results lack statistical clarity. The author needs to find out the key findings from the manuscript and report it in the result section precisely.

The author mentioned that "Multivariable analysis revealed caregiver age and awareness of poor hygiene as a risk factor for trachoma and were also significantly associated with TF." This needs more clarity in writing and grammatical correction.

Introduction

The Introduction is relevant and generally informative. The knowledge gap and specific rationale for conducting this study could be more explicitly stated.

Methods

The manuscript raises several important methodological concerns that need to be addressed to strengthen its scientific rigor. First, the study appears to employ a cross-sectional design; however, this is not explicitly stated. Therefore, the inherent limitation of this design in establishing causal relationships should be acknowledged and discussed more explicitly.

The sample size calculation seems incomplete. While the authors state that Cochran's formula was used, key parameters such as the CI and margin of error are not specified, making it difficult to assess the appropriateness and reproducibility of the sample size calculation. The multistage sampling approach seems appropriate for this study. However, the use of the random walk method for household selection within villages raises concerns about potential selection bias. The author mentioned about 18 clusters; however it is unclear whether equal or proportional allocation was used.

The author includes key variables relevant to trachoma transmission. However, the author should clearly describe the key exposure and outcome variables. Like, facial cleanliness is not clearly operationalized, and it is unclear whether established indicators were used, raising concerns about measurement validity. Similarly,

environmental variables such as water source and sanitation are not defined using standardized classifications, which lacks clarity.

The statistical analysis section lacks sufficient detail to assess the robustness of the findings. The manuscript does not clearly describe the statistical models, the approach to adjusting for potential confounders, or whether clustering at the household or community level was accounted for in the analysis. These exclusion about confounding and clustering may lead to biased estimates and incorrect inferences.

Results

The Results section presents findings that are relevant to the study objectives, however, the presentation of results appears to be overly descriptive, with insufficient emphasis on key findings. The manuscript would benefit from clearer structuring, with a logical progression from descriptive statistics to inferential analysis. There is limited evidence that confounding factors have been adequately controlled for in the analysis. Given the multifactorial nature of trachoma, failure to adjust for potential confounders may bias the results. Also, the handling of clustered data is not mentioned in the results section. Given the nature of multistage sampling design mentioned in the sampling part, it is important to account for clustering. If not properly addressed, this could lead to underestimation of standard errors and overstatement of statistical significance.

Discussion

The discussion addresses an important public health issue and attempts to interpret the findings within the broader context of trachoma control. While the section includes the necessary elements, the overall flow and logical connections are weak. Ideas are presented in a fragmented manner, with abrupt transitions between findings and limited connections between paragraphs. In some areas, the text reads more like a repetition of results rather than an interpretation, which reduces analytical depth. Although a limitations section is included, several important methodological limitations and potential biases have been overlooked. A more explicit and structured discussion of study limitations is needed. Recommendations provided by the author are general and not clearly linked to the study findings.

General comments: The authors should carefully review the manuscript to improve clarity including correcting grammatical errors, ensuring consistency in referencing style, and breaking up long or complex sentences.

The manuscript addresses an important public health issue and provides context specific thoughts, however, the methodological limitations and unclear reporting weaken the robustness of the findings. Significant improvements in study design justification, analytical depth, and writing clarity are needed before it is suitable for publication.
