# Supplementary material for: Facial cleanliness, environmental factors, and the burden of trachoma in a pastoral conflict area of Tiaty East, Baringo County, Kenya
Source: PLOS Glob Public Health. 2026 Oct 27;6(10):e0007534. doi: 10.1371/journal.pgph.0007534 (PMC13712619; doi:10.1371/journal.pgph.0007534)
Supplement: Supplementary file 2 [file pgph.0007534.s001.docx]

# **S1 Appendix: Supplementary Tables**

## **Table A: Variance inflation factors (VIFs) for variables included in the multivariable model**

| **Variable** | **Maximum VIF** |
| --- | --- |
| Caregiver age | 5.54 |
| Household income | 3.16 |
| Time to water source | 1.15 |
| Presence of flies around the household | 1.18 |
| Facial cleanliness indicator | 1.40 |
| Caregiver knowledge of cause of trachoma | 8.19 |
| **Mean VIF** | **3.96** |

## **Table B: ROC analysis for the final multivariable model**

| **Diagnostic Measure** | **Estimate** | **95% CI** |
| --- | --- | --- |
| Area under the ROC curve (AUC) | 0.870 | 0.772–0.967 |

## **Table C: Sensitivity analysis- Odds ratios (OR) and 95% confidence intervals (CI) for predictors of trachomatous inflammation folliculitis across alternative model specifications**

| **Variable** | **Model-based SE** | | **Mixed-effects logistic** | | **Cluster bootstrap** | | **Independence correlation** | |
| --- | --- | --- | --- | --- | --- | --- | --- | --- |
|  | **OR (95% CI)** | **p-value** | **OR (95% CI)** | **p-value** | **OR (95% CI)** | **p-value** | **OR (95% CI)** | **p-value** |
| ***Age of primary caregiver (Ref: ≤20 years)*** | | | | | | | | |
| 21–30 years | 0.09 (0.01–0.92) | 0.042 | 0.08 (0.01–0.88) | 0.039 | 0.09 (0.04–0.19) | 0.000 | 0.08 (0.02–0.43) | 0.003 |
| 31–40 years | 0.26 (0.03–2.36) | 0.231 | 0.26 (0.03–2.37) | 0.233 | 0.26 (0.07–1.03) | 0.055 | 0.26 (0.07–0.96) | 0.043 |
| ≥40 years | 0.16 (0.02–1.55) | 0.114 | 0.16 (0.02–1.56) | 0.115 | 0.16 (0.08–0.32) | <0.001 | 0.16 (0.03–0.85) | 0.032 |
| ***Household income (Ref: ≤$7.75)*** | | | | | | | | |
| ≥7.76 - 38.75 | 0.62 (0.07–5.80) | 0.673 | 0.63 (0.07–5.96) | 0.687 | 0.62 (0.19–2.02) | 0.426 | 0.63 (0.15–2.74) | 0.538 |
| ≥38.76 - 77.51 | ***Predicts failure perfectly | | | | | | | |
| ≥77.52 | 1.17 (0.04–31.44) | 0.925 | 1.19 (0.04–32.30) | 0.920 | 1.17 (0.24–5.83) | 0.847 | 1.19 (0.10–14.06) | 0.893 |
| ***Time to fetch water (Ref: >1 hour)*** | | | | | | | | |
| 30 min to 1 hour | 0.06 (0.01–0.71) | 0.026 | 0.06 (0.01–0.74) | 0.028 | 0.06 (0.02–0.21) | <0.001 | 0.06 (0.00–1.03) | 0.053 |
| ***Poor personal hygiene (Ref: No)*** | | | | | | | | |
| Yes | 3.80 (0.33–44.29) | 0.287 | 3.81 (0.32–45.91) | 0.293 | 3.80 (1.04–13.80) | 0.043 | 3.81 (1.29–11.26) | 0.016 |
| ***Dirty environment (Ref: No)*** | | | | | | | | |
| Yes | 0.58 (0.10–3.51) | 0.556 | 0.57 (0.09–3.48) | 0.546 | 0.58 (0.23–1.46) | 0.251 | 0.57 (0.21–1.54) | 0.270 |
| ***Knowledge about cause of trachoma (Ref: Witchcraft/Curse)*** | | | | | | | | |
| Flies | 0.16 (0.00–11.06) | 0.393 | 0.16 (0.00–11.01) | 0.397 | 0.16 (0.07–0.36) | <0.001 | 0.16 (0.02–1.03) | 0.054 |
| Bacterial infection | 0.12 (0.00–14.52) | 0.383 | 0.12 (0.00–14.80) | 0.386 | 0.12 (0.02–0.67) | 0.016 | 0.12 (0.01–2.65) | 0.178 |

**Note:** All models include the same covariates. Model-based SE = GEE with exchangeable working correlation and model-based standard errors; Mixed-effects = subject-specific random-effects logistic regression; Cluster bootstrap = GEE with exchangeable correlation and cluster-bootstrapped standard errors (500 replications); Independence = GEE with independence working correlation and robust standard errors. OR = odds ratio; CI = confidence interval; Ref = reference category
