# Supplementary material for: Facial cleanliness, environmental factors, and the burden of trachoma in a pastoral conflict area of Tiaty East, Baringo County, Kenya
Source: PLOS Glob Public Health. 2026 Oct 27;6(10):e0007534. doi: 10.1371/journal.pgph.0007534 (PMC13712619; doi:10.1371/journal.pgph.0007534)
Supplement: Supplementary file 4 [file pgph.0007534.s005.docx]

22^nd^ May 2026

The Editorial Board

**PLOS Global Public Health**

**Re: Manuscript PGPH-D-25-03058 – *“Facial cleanliness, environmental factors, and the burden of trachoma in a pastoral conflict area of Tiaty East Baringo County, Kenya”***

Dear Editor and Reviewers,

Thank you for your thorough and constructive assessment of our manuscript. The comments have helped us to substantially strengthen the scientific rigour, clarity, and completeness of the paper. We have carefully addressed each comment and outline our responses below. All changes to the manuscript are indicated using tracked changes in the revised document.

For the convenience of the reviewers, the comments and corresponding revisions are listed below: reproduced in full, followed by our point-by-point response and a description of the corresponding revision made to the manuscript.

**Response to Reviewer 1**

## Introduction

| **Comment 1.1** | The paper mentioned “Baringo County offers a unique context …”. While the introduction provides a few features of Baringo County’s uniqueness, it did not provide any comparison of how the other counties are different. Many other counties (e.g., Turkana, Marsabit, Kajiado) also experience similar vulnerabilities. Please explain why Baringo County is unique compared to other similar contexts/counties. |
| --- | --- |
| **Response** | We appreciate this valuable comment. The introduction has been improved by providing comparisons with similar contexts in the country as suggested. We have included comparisons on MDA coverage and F&E from published studies. Lines 101, 112-117 |

| **Comment 1.2** | The introduction mentions a TF prevalence of 3.02% in 2023. In that case, why do we need another study that looks for the prevalence of TF? |
| --- | --- |
| **Response** | We have clarified the need for another prevalence study. While the 2023 survey was conducted at the EU level, a previous study has shown gaps in Tiaty East MDA coverage, which could lead to pockets of infection in the area. Sub-EU level prevalence data is therefore important to help guide targeted interventions. Lines 103-106 |

| **Comment 1.3** | Why did you specifically select Tiaty East? Is there anything unique about this sub-county? |
| --- | --- |
| **Response** | Tiaty East is unique due to the consistently below-target MDA coverage with no ongoing MDA activities and active surveillance. This is now explicitly mentioned in the manuscript. Lines 100-106 |

| **Comment 1.4** | Please clarify what you mean by household prevalence. |
| --- | --- |
| **Response** | We thank the reviewer for this clarification request. On reflection, we acknowledge that the term 'household prevalence' is imprecise, as TF is measured in individual children aged 1–9 years rather than in households. This has been duly addressed in the manuscript. Lines 123-124 |

| **Comment 1.5** | Briefly describe what the few other studies found that examined household F&E aspects in marginalized communities, so we can understand where this study might contribute. |
| --- | --- |
| **Response** | This addition is accepted and included in the manuscript, as also mentioned in comment 1.2. Lines 112-117 |

## Materials and Methods

| **Comment 1.6** | Ethics statement should start with the informed consent process; after that, provide the description of approvals. |
| --- | --- |
| **Response** | Accepted. The Ethics Statement has been restructured so that the informed consent process is described first, followed by the institutional approvals. Line 129 |

| **Comment 1.7** | Move “Data was collected between 19th – 26th February 2025” to Data Collection and Management. |
| --- | --- |
| **Response** | Accepted. The data collection dates have been moved to the opening sentence of the Data Collection and Management sub-section. Line 186 |

| **Comment 1.8** | The calculation method is not robust. The paper used a TF prevalence of 5.9%, but the introduction mentions 3.02% in 2023. Also, justification for the design effect of 2.0 is needed, as well as justification for the 10% attrition assumption. Provide a more detailed version including power calculation. |
| --- | --- |
| **Response** | The prevalence of 5.9% was used instead of the reported 3.02% in the impact survey of 2023 as the sample size calculation had been done based on a larger prevalence figure from several trachoma-endemic communities in Kenya. This estimate was chosen as it was considered to be a more conservative estimate, which would yield a larger sample size and consequently higher statistical precision. We have also revised the section to include parameters used in sample size calculation, and justification for the assumed design effect and non-response. Lines 159-166 |

| **Comment 1.9** | The study did not reach the adequate sample of 190. Please mention how you addressed that. Add this in the limitation section if required. |
| --- | --- |
| **Response** | The study reached 178 of the planned 190 households (94% of target households) with only 12 households (6%) not reached, which our study meets. This response rate meets the 85% considered adequate for cross-sectional studies and therefore did not impact the ability to estimate TF prevalence, especially since our ultimate sample were children aged 1-9years in these households. This could, however, have slightly underestimated the precision in detecting associations, especially with few TF cases. This restriction is noted in the manuscript limitation section. Lines 218-221, 473-475 |

| **Comment 1.10** | Sample size is calculated based on households, but TF is calculated for children 1–9. The TF prevalence among children may differ from what the household-based estimate considers. Please explain how you addressed this. |
| --- | --- |
| **Response** | We acknowledge that the sample size was calculated using households as the sampling unit, whereas TF prevalence was estimated at the child level. Households were selected because the principal exposures were measured at the household and caregiver levels, and all eligible children aged 1–9 years within sampled households were examined. Consequently, the effective sample size for the prevalence estimate was the total number of examined children (n = 279). Lines 167-168, 218-219 |

| **Comment 1.11** | Final model included only statistically significant variables, which might introduce selection bias. I highly recommend running the model considering all the relevant confounders. |
| --- | --- |
| **Response** | We agree that epidemiological relevant adjustment is important. Due to a small number of observed TF cases, however, it would have been difficult to include many covariates in the model, as we would run the risk of model overfitting and unstable estimates. We therefore adopted a two-stage approach: first, variables were assessed in domain-specific models, and those achieving statistical significance were retained and carried forward into the final multivariable model. The exploratory nature of the regression analyses and this analytical approach have been explicitly recognised as limitations. Lines 473-475 |

| **Comment 1.12** | The GEE model does not specify the clustering level, correlation structure, and whether standard errors were robust. |
| --- | --- |
| **Response** | Accepted. An exchangeable working correlation structure was specified, assuming that all children within the same household had the same pairwise correlation in TF status. This structure was chosen because the data were cross-sectional and clustered within households without any inherent ordering. Line 208-209 |

| **Comment 1.13** | Given the low TF prevalence (<5%), please show model diagnostic results (multicollinearity checks, model fit, sensitivity analysis) in supplementary files*.* |
| --- | --- |
| **Response** | Model diagnostic results have now been submitted as a supplementary file. Sensitivity analyses using mixed-effects logistic regression, cluster bootstrap, and alternative GEE correlation structures yielded effect estimates that were generally consistent in direction and magnitude with the primary GEE model. Differences in statistical significance were observed for some variables due to the small number of TF cases and resulting wide confidence intervals.  Overall, these findings support the robustness of the main results. Lines 267-272 |

| **Comment 1.14** | TF was measured among children 1–9, but many predictors are household/caregiver level. Provide a conceptual framework of how children-caregiver-different predictors are related. |
| --- | --- |
| **Response** | This is a valuable suggestion and is now provided as *Fig. 2.* Line 215 |

| **Comment 1.15** | Clarify when the data collectors observed facial cleanliness (e.g., if it was during play time). |
| --- | --- |
| **Response** | We have added a clarifying statement to the Data Collection section noting that facial cleanliness was assessed by trained research assistants at the time of the household visit(between 7am and 6pm). We acknowledge that the timing of observation relative to the child’s activities (play, meal, bath, etc) may influence findings. However, transient facial uncleanliness may still be epidemiologically relevant, it can attract eye-seeking flies implicated in trachoma transmission. Line 191 |

## Results

| **Comment 1.16** | Monthly household income – show in USD for a larger audience. |
| --- | --- |
| **Response** | Accepted. USD equivalents have been added throughout the Results. A footnote clarifying the exchange rate used has been added to the table. Lines 236 |

| **Comment 1.17** | “Availability of hygiene and sanitation infrastructure is important for good hygiene practices” is not important in the result section. Please delete. |
| --- | --- |
| **Response** | Accepted. This interpretive sentence has been removed from the Results section. |

| **Comment 1.18** | Tables 4–6 can be merged into one table. |
| --- | --- |
| **Response** | Accepted. To preserve readability, we have structured the tables with clear sections. Lines 264-265 |

| **Comment 1.19** | Several variables are marked as “*** omitted due to collinearity” or “predicts failure perfectly”. Please briefly explain how this affected interpretation or model selection. |
| --- | --- |
| **Response** | This has been mentioned as a preamble under the “Factors associated with the prevalence of Trachoma Folliculitis” section. Lines 267-274 |

| **Comment 1.20** | Please avoid using “Risk factor for trachoma” or “Protective against TF” as these seem like causal claims; instead, describe them as associations. |
| --- | --- |
| **Response** | Accepted. All causal language has been replaced with associative framing throughout the manuscript. |

## Discussion

| **Comment 1.21** | The paper mentioned “prevalence rate” but you did not calculate a rate. It should be “prevalence”. Also, without a 95% CI the statement about 5.02% can be misleading. |
| --- | --- |
| **Response** | Accepted. All instances of “trachoma prevalence rate” have been corrected to “TF prevalence” throughout the manuscript. The 95% is now presented alongside the prevalence. Lines 334-338 |

| **Comment 1.22** | Comparisons with prior impact surveys should be framed cautiously given differences in survey design. References to ‘pockets of active trachoma’ should be presented as hypotheses rather than conclusions. |
| --- | --- |
| **Response** | Accepted. The relevant passage has been revised and presented as a possibility rather than a conclusion. Lines 340-343 |

| **Comment 1.23** | The interpretation occasionally exceeds what can be supported by the cross-sectional design. Greater emphasis on uncertainty, model limitations, and the exploratory nature of associations would strengthen the discussion. |
| --- | --- |
| **Response** | We have revised the Discussion to consistently frame associations as exploratory. These suggested points have also been consolidated in the Limitations section. Lines 345-347, 368, 413-414, 430-435 |

## Conclusion

| **Comment 1.24** | Make the conclusion result-based and very specific. The sentence “Ultimately, trachoma elimination is more than a disease control milestone …” is not based on your results. |
| --- | --- |
| **Response** | Accepted. The conclusion section has been substantially revised to be results-based and very specific. Lines 430-452 |

## Limitations

| **Comment 1.25** | Describe low prevalence and potential issue with statistical power; state that the TF estimate is near the 5% WHO threshold; mention cross-sectional design limitations; acknowledge the achieved sample was below target. |
| --- | --- |
| **Response** | We have revised the Limitation section to include cross-sectional design limitations and the effect of low prevalence on precision in multivariable models as advised. We, however, have addressed that the sample achieved met the 85% threshold required in cross-sectional studies in the results section as responded under comment 1.9. Lines 454-477 |

**Response to Reviewer 2**

## Abstract

| **Comment 2.1** | The objective is not explicitly written in the abstract. |
| --- | --- |
| **Response** | An explicit objective sentence has been provided in the abstract on Line 25-27 |

| **Comment 2.2** | In the results part of the abstract, regression analysis is poorly articulated; without these, the results lack statistical clarity. “Multivariable analysis revealed caregiver age and awareness of poor hygiene as a risk factor for trachoma and were also significantly associated with TF.” This needs more clarity and grammatical correction. |
| --- | --- |
| **Response** | Thank you for this meticulous observation. The abstract results sentence has been rewritten to clearly articulate the multivariable findings while attempting to keep the abstract precise. Line 36-41 |

## Introduction

| **Comment 2.3** | The knowledge gap and specific rationale for conducting this study could be more explicitly stated. |
| --- | --- |
| **Response** | This is also an issue noted by reviewer 1. We have revised the section to justify our study. Lines 100-106 |

## Methods

| **Comment 2.4** | The cross-sectional design is not explicitly stated, and the inherent limitation in establishing causal relationships should be acknowledged. |
| --- | --- |
| **Response** | We have stated this explicitly in the Study design section. The limitations for this design has also been acknowledged in the Limitation section. Lines 154, 455-456 |

| **Comment 2.5** | The sample size calculation seems incomplete. Key parameters such as the CI and margin of error are not specified. |
| --- | --- |
| **Response** | Accepted. The Sample Size Calculation section has been expanded to include all parameters. The prevalence estimate used (5.9%), the design effect (2.0), and the attrition rate (10%) are all now stated with justification. Lines 159-160 |

| **Comment 2.6** | The use of the random walk method raises concerns about potential selection bias. It is unclear whether equal or proportional allocation was used across 18 clusters. |
| --- | --- |
| **Response** | We have added clarification to the Sampling section. Proportional allocation was used relative to village population size. The random walk method was used as a pragmatic approach in the field, where household registers were unavailable. We acknowledge the potential for selection bias and have added this to the Limitations section. Lines 179-183, 461-468 |

| **Comment 2.7** | Facial cleanliness is not clearly operationalized, and environmental variables are not defined using standardized classifications. |
| --- | --- |
| **Response** | This has been addressed in the Data Collection and Management section. Lines 188-194 |

| **Comment 2.8** | The statistical analysis section lacks sufficient detail on models, confounders, and clustering. These exclusions may lead to biased estimates. |
| --- | --- |
| **Response** | We agree that epidemiological relevant adjustment is important. Due to a small number of observed TF cases, however, it would have been difficult to include many covariates in the model, as we would run the risk of model overfitting and unstable estimates. We therefore adopted a two-stage approach: first, variables were assessed in domain-specific models, and those achieving statistical significance were retained and carried forward into the final multivariable model. The exploratory nature of the regression analyses and this analytical approach have been explicitly recognised as limitations. Lines 267-274, 473-475 |

## Results

| **Comment 2.9** | The presentation is overly descriptive with insufficient emphasis on key findings. The handling of clustered data is not mentioned in the results. |
| --- | --- |
| **Response** | We have refined the Data Analysis section to explicitly state that the GEE model accounted for clustering at the village level (Lines 207-210). We emphasized the key findings before presenting supporting tables. |

## Discussion

| **Comment 2.10** | The overall flow and logical connections are weak. Ideas are fragmented, and the text reads more like a repetition of results than an interpretation. Recommendations are general and not linked to study findings. |
| --- | --- |
| **Response** | Thank you for this candid assessment. The Discussion has been revised to open each paragraph with an interpretive statement rather than a restatement of results. Transitions have been made between paragraphs, especially in the subsection of Environmental Improvement, to create a logical narrative. The Conclusion and Recommendations section has been substantially revised to link them to specific study findings. Lines 444-452 |

| **Comment 2.11** | Several important methodological limitations have been overlooked. A more explicit and structured discussion of study limitations is needed. |
| --- | --- |
| **Response** | Accepted. The Limitations section has been comprehensively rewritten and expanded (see also our response to Reviewer 1, Comment 1.25). It now addresses all major limitations in a structured manner. Lines 454-477 |

## General Comments

| **Comment 2.12** | The authors should carefully review the manuscript to improve clarity including correcting grammatical errors, ensuring consistency in referencing style, and breaking up long or complex sentences. |
| --- | --- |
| **Response** | Accepted. The entire manuscript has been reviewed for grammatical accuracy, sentence clarity, and consistency in referencing. Long or complex sentences have been broken up throughout. Specific corrections are visible in the tracked-changes version of the revised manuscript. |

We believe these revisions substantially address the concerns raised by both reviewers and strengthen the manuscript considerably. We are grateful for the opportunity to improve this work and look forward to the Editor’s further evaluation.

Yours sincerely,

**Loise Wanjira Njogu**

On behalf of all co-authors
