# Supplementary material for: Facial cleanliness, environmental factors, and the burden of trachoma in a pastoral conflict area of Tiaty East, Baringo County, Kenya
Source: PLOS Glob Public Health. 2026 Oct 27;6(10):e0007534. doi: 10.1371/journal.pgph.0007534 (PMC13712619; doi:10.1371/journal.pgph.0007534)
Supplement: Supplementary file 5 [file pgph.0007534.s002.docx]

Introduction

- The paper mentioned “Baringo County offers a unique context with an intersection of factors that could potentially impact on the effectiveness of F&E as well as exacerbating the disease, hence a good context for the study”. While the introduction provides a few features of Baringo County’s uniqueness (e.g. nomadic pastoralism, conflict-and insecurity, poor WASH infrastructure, persistent trachoma transmission despite mass drug administration), it did not provide any comparison of how the other counties are different. The way I understand it, many other counties (e.g. Turkana, Marsabit, Kajiado) also experience the similar vulnerabilities and contexts. Please explain why Baringo County is unique compared to other similar contexts/counties.
- The introduction mentioned “While Baringo County has seen a significant reduction of TF from 34.3% in 2011 to 3.02% in 2023 due to intensive MDA interventions, limited access to the resident population due to insecurity and nomadic lifestyle poses a challenge in effectively reaching members of the community through MDA(16)” which I think means the county has the prevalence of TF available in 2023. In that case, why do we need another study that looks for the prevalence of TF?
- Why did you specifically select ‘Tiaty East’ of the Baringo County? Is there anything unique about this?
- Please clarify what you mean by household prevalence.
- Also, you might want to briefly describe what the few other studies found that examined household F&E aspects in marginalized communities. In that case, we can understand where this study might contribute.

Materials and Methods

- Ethics statement should start with the informed consent process; after that mentioned provide the description on approvals.
- Move the following to data collection and management “Data was collected between 19th – 26th February 2025”

Study area:

- Please clarify why the Baringo County and, in partular Tiaty East Sub-County was selected.

Sample size calculation and sampling technique

- The calculation method is not robust; e.g., the paper used a TF prevalence of 5.9%, but in the introduction, it mentioned the TF prevalence of 3.02% in 2023 in Baringo County. Also, the study selected the sub-EU and post-MDA setting and if it’s prevalence is different, this affects the overall sample size
- Need justification on why the design effect of 2.0 was selected
- Please clarify why the assumed 10% attrition in Baringo County’s context
- Provide a more detailed version of the sample size calculation, including power calculation
- Also, the study did not reach the adequate (as mentioned within existing sample size calculation) sample of 190; it has a greater impact on statistical power and precision of the results; Please mention how you addressed that. Add this in the limitation section if required.
- Sample size is calculated based on the households, but TF is calculated for children 1-9; the TF prevalence among children may differ from what the HH-based estimate considers. Please explain how you addressed this.

Data analysis

- Final model included only statistically significant variables, which might miss important confounders (not wrong statistically, but extremely important if we consider the epidemiological concepts); this introduces selection bias. I highly recommend running the model, considering all the relevant confounders.
- The GEE model does not specify the clustering level (household?), correlation structure, and whether standard errors were robust. Please consider mentioning these.
- The prevalence of TF is very low (less than 5%); to ensure the model is not overfitting, please show the model diagnostic results (including multicollinearity checks, model fit, sensitivity analysis); put the results in supplementary files with the manuscript

- Overall method: TF was measured among children 1-9 but many of the predictors are household/caregiver level. Provide a conceptual framework of how children-caregiver-different predictors are related/ it’s impacts.

- also clarify when the data collectors observed the facial cleanliness. E.g. If it was during play time!

Result

- Socio demographic and economic characteristics: “monthly household income 192 ranging from KES 1001-4999.”; also show in USD for a larger audience
- In Hygiene and sanitation facilities section, the following sentence is not important in the result section “Availability of hygiene and sanitation infrastructure is important for good hygiene practices”. You can delete this from here
- Table 4-6 can be merged into one table
- Several variables are marked as “*** omitted due to collinearity” or “predicts failure perfectly” which refers to potential model instability; please briefly explain how this affected interpretation or model selection.
- Please avoid using “Risk factor for trachoma” or “Protective against TF” as these seem like causal claims; instead, describe them as associations.

Discussion

- The paper mentioned prevalence rate; but you did not calculate rate; it should be prevalence; you also mentjoned “This study established a trachoma prevalence rate of 5.02% in Tiaty East(a sub 298 EU), which is slightly higher than the WHO trachoma elimination target of <5.00% (4).”. However, without a 95% CI, the statement can be misleading.
- Comparisons with prior impact surveys should be framed cautiously given differences in survey design, and references to “pockets of active trachoma” and targeted interventions should be presented as hypotheses rather than conclusions derived directly from cross-sectional data.
- Overall, the interpretation occasionally exceeds what can be supported by the cross-sectional design and limited number of TF events, particularly when drawing causal inferences or reconciling null associations for F&E variables. Greater emphasis on uncertainty, model limitations, and the exploratory nature of several associations would strengthen the balance and credibility of the discussion

Conclusion:

- Make the conclusion result based and very specific. E.g. the following sentence is not based on your result “Ultimately, trachoma elimination is more than a disease control milestone, it is a matter of equity and human rights, preventing avoidable blindness and enabling communities to thrive free from the burden of a preventable disease.”

Limitation

- Describe low prevalence of TF and potential issue with statistical power
- Explicitly state that the TF estimate is near the 5% WHO threshold, and that confidence intervals overlap the cutoff, limiting certainty.
- Mention the issue with cross section design: Clearly state that temporal relationships cannot be established. Avoid implying that poor F&E conditions caused TF persistence.
- Briefly acknowledge that the achieved sample size was slightly below the calculated target, with potential implications for precision.
