# Supplementary material for: Facial cleanliness, environmental factors, and the burden of trachoma in a pastoral conflict area of Tiaty East, Baringo County, Kenya
Source: PLOS Glob Public Health. 2026 Oct 27;6(10):e0007534. doi: 10.1371/journal.pgph.0007534 (PMC13712619; doi:10.1371/journal.pgph.0007534)
Supplement: Peer review history

# Author comment: Author response to Decision Letter 0

**DOI:** 10.1371/journal.pgph.0007534.r001

**Transfer Alert**

This paper was transferred from another journal. As a result, its full editorial history (including decision letters, peer reviews and author responses) may not be present.

11 Oct 2025

# Peer review: Decision Letter 0

**DOI:** 10.1371/journal.pgph.0007534.r002

**Authors:** Xin Sun

8 Apr 2026

PGPH-D-25-03058

Facial cleanliness, environmental factors and the burden of trachoma in a pastoral conflict area of Tiaty East Baringo County, Kenya

PLOS Global Public Health

Dear Dr. Njogu,

Thank you for submitting your manuscript to PLOS Global Public Health. After careful consideration, we feel that it has merit but does not fully meet PLOS Global Public Health’s publication criteria as it currently stands. Therefore, we invite you to submit a revised version of the manuscript that addresses the points raised during the review process.

Please submit your revised manuscript by May 23 2026 11:59PM. If you will need more time than this to complete your revisions, please reply to this message or contact the journal office at globalpubhealth@plos.org. When you're ready to submit your revision, log on to https://www.editorialmanager.com/pgph/ and select the 'Submissions Needing Revision' folder to locate your manuscript file.

Please include the following items when submitting your revised manuscript:

- A letter that responds to each point raised by the editor and reviewer(s). You should upload this letter as a separate file labeled 'Response to Reviewers'.
- A marked-up copy of your manuscript that highlights changes made to the original version. You should upload this as a separate file labeled 'Revised Manuscript with Track Changes'.
- An unmarked version of your revised paper without tracked changes. You should upload this as a separate file labeled 'Manuscript'.

Guidelines for resubmitting your figure files are available below the reviewer comments at the end of this letter.

As the corresponding author, your ORCID iD is verified in the submission system and will appear in the published article. PLOS supports the use of ORCID, and we encourage all coauthors to register for an ORCID iD and use it as well. Please encourage your coauthors to verify their ORCID iD within the submission system before final acceptance, as unverified ORCID iDs will not appear in the published article. *Only*  the individual author can complete the verification step; PLOS staff *cannot*  verify ORCID iDs on behalf of authors.

We look forward to receiving your revised manuscript.

Kind regards,

Xin Sun, PhD

Staff Editor

PLOS Global Public Health

**Journal Requirements:**

1.  Please amend your detailed Financial Disclosure statement. This is published with the article. It must therefore be completed in full sentences and contain the exact wording you wish to be published.

i. Please clarify all sources of financial support for your study. List the grants, grant numbers, and organizations that funded your study, including funding received from your institution. Please note that suppliers of material support, including research materials, should be recognized in the Acknowledgements section rather than in the Financial Disclosure.

ii. State the initials, alongside each funding source, of each author to receive each grant. For example: "This work was supported by the National Institutes of Health (####### to AM; ###### to CJ) and the National Science Foundation (###### to AM)."

iii. State what role the funders took in the study. If the funders had no role in your study, please state: “The funders had no role in study design, data collection and analysis, decision to publish, or preparation of the manuscript.”

iv. If any authors received a salary from any of your funders, please state which authors and which funders.

2. Some material included in your submission may be copyrighted. According to PLOS’s copyright policy, authors who use figures or other material (e.g., graphics, clipart, maps) from another author or copyright holder must demonstrate or obtain permission to publish this material under the Creative Commons Attribution 4.0 International (CC BY 4.0) License used by PLOS journals. Please closely review the details of PLOS’s copyright requirements here: PLOS Licenses and Copyright. If you need to request permissions from a copyright holder, you may use PLOS's Copyright Content Permission form.

Please respond directly to this email or email the journal office and provide any known details concerning your material's license terms and permissions required for reuse, even if you have not yet obtained copyright permissions or are unsure of your material's copyright compatibility.

Potential Copyright Issues:

a. Figure 1: please (a) provide a direct link to the base layer of the map (i.e., the country or region border shape) and ensure this is also included in the figure legend; and (b) provide a link to the terms of use / license information for the base layer image or shapefile. We cannot publish proprietary or copyrighted maps (e.g. Google Maps, Mapquest) and the terms of use for your map base layer must be compatible with our CC-BY 4.0 license.

Note: if you created the map in a software program like R or ArcGIS, please locate and indicate the source of the basemap shapefile onto which data has been plotted.

If your map was obtained from a copyrighted source please amend the figure so that the base map used is from an openly available source. Alternatively, please provide explicit written permission from the copyright holder granting you the right to publish the material under our CC-BY 4.0 license.

Please note that the following CC BY licenses are compatible with PLOS license: CC BY 4.0, CC BY 2.0 and CC BY 3.0, meanwhile such licenses as CC BY-ND 3.0 and others are not compatible due to additional restrictions.

If you are unsure whether you can use a map or not, please do reach out and we will be able to help you. The following websites are good examples of where you can source open access or public domain maps:

* U.S. Geological Survey (USGS) - All maps are in the public domain. (http://www.usgs.gov)

* PlaniGlobe - All maps are published under a Creative Commons license so please cite “PlaniGlobe, http://www.planiglobe.com, CC BY 2.0” in the image credit after the caption. (http://www.planiglobe.com/?lang=enl)

* Natural Earth - All maps are public domain. (http://www.naturalearthdata.com/about/terms-of-use/)

If the reviewer comments include a recommendation to cite specific previously published works, please review and evaluate these publications to determine whether they are relevant and should be cited. There is no requirement to cite these works unless the editor has indicated otherwise.

**Additional Editor Comments (if provided):**

[Note: HTML markup is below. Please do not edit.]

Reviewers' comments:

Reviewer's Responses to Questions

**Comments to the Author**

1. Does this manuscript meet PLOS Global Public Health’s publication criteria?

Reviewer #1: Yes

Reviewer #2: No

**********

2. Has the statistical analysis been performed appropriately and rigorously?

Reviewer #1: No

Reviewer #2: No

**********

3. Have the authors made all data underlying the findings in their manuscript fully available (please refer to the Data Availability Statement at the start of the manuscript PDF file)?

The PLOS Data policy

Reviewer #1: Yes

Reviewer #2: Yes

**********

4. Is the manuscript presented in an intelligible fashion and written in standard English?

Reviewer #1: Yes

Reviewer #2: No

**********

**Reviewer #1:**  This paper has clear public health relevance. However, clearer justification of study context, stronger methods, cautious interpretation, and better handling of low TF prevalence are needed. I have provided the detailed review in the attachment

**Reviewer #2:**  Manuscript title: Facial cleanliness, environmental factors and the burden of trachoma in a pastoral conflict area of Tiaty East Baringo County, Kenya

Manuscript Number PGPH-D-25-03058

Comments reviewer:

Abstract

The abstract highlights the global burden of Trachoma, emphasizing the status of the leading infectious cause of preventable blindness. However, the objective is not explicitly written. It seems unclear for the reader.

In the results part, the author more likely focused on descriptive analysis, while regression analysis are poorly articulated; without these, the results lack statistical clarity. The author needs to find out the key findings from the manuscript and report it in the result section precisely.

The author mentioned that “Multivariable analysis revealed caregiver age and awareness of poor hygiene as a risk factor for trachoma and were also significantly associated with TF.” This needs more clarity in writing and grammatical correction.

Introduction

The Introduction is relevant and generally informative. The knowledge gap and specific rationale for conducting this study could be more explicitly stated.

Methods

The manuscript raises several important methodological concerns that need to be addressed to strengthen its scientific rigor. First, the study appears to employ a cross-sectional design; however, this is not explicitly stated. Therefore, the inherent limitation of this design in establishing causal relationships should be acknowledged and discussed more explicitly.

The sample size calculation seems incomplete. While the authors state that Cochran’s formula was used, key parameters such as the CI and margin of error are not specified, making it difficult to assess the appropriateness and reproducibility of the sample size calculation. The multistage sampling approach seems appropriate for this study. However, the use of the random walk method for household selection within villages raises concerns about potential selection bias. The author mentioned about 18 clusters; however it is unclear whether equal or proportional allocation was used.

The author includes key variables relevant to trachoma transmission. However, the author should clearly describe the key exposure and outcome variables. Like, facial cleanliness is not clearly operationalized, and it is unclear whether established indicators were used, raising concerns about measurement validity. Similarly, environmental variables such as water source and sanitation are not defined using standardized classifications, which lacks clarity.

The statistical analysis section lacks sufficient detail to assess the robustness of the findings. The manuscript does not clearly describe the statistical models, the approach to adjusting for potential confounders, or whether clustering at the household or community level was accounted for in the analysis. These exclusion about confounding and clustering may lead to biased estimates and incorrect inferences.

Results

The Results section presents findings that are relevant to the study objectives, however, the presentation of results appears to be overly descriptive, with insufficient emphasis on key findings. The manuscript would benefit from clearer structuring, with a logical progression from descriptive statistics to inferential analysis. There is limited evidence that confounding factors have been adequately controlled for in the analysis. Given the multifactorial nature of trachoma, failure to adjust for potential confounders may bias the results. Also, the handling of clustered data is not mentioned in the results section. Given the nature of multistage sampling design mentioned in the sampling part, it is important to account for clustering. If not properly addressed, this could lead to underestimation of standard errors and overstatement of statistical significance.

Discussion

The discussion addresses an important public health issue and attempts to interpret the findings within the broader context of trachoma control. While the section includes the necessary elements, the overall flow and logical connections are weak. Ideas are presented in a fragmented manner, with abrupt transitions between findings and limited connections between paragraphs. In some areas, the text reads more like a repetition of results rather than an interpretation, which reduces analytical depth. Although a limitations section is included, several important methodological limitations and potential biases have been overlooked. A more explicit and structured discussion of study limitations is needed. Recommendations provided by the author are general and not clearly linked to the study findings.

General comments: The authors should carefully review the manuscript to improve clarity including correcting grammatical errors, ensuring consistency in referencing style, and breaking up long or complex sentences.

The manuscript addresses an important public health issue and provides context specific thoughts, however, the methodological limitations and unclear reporting weaken the robustness of the findings. Significant improvements in study design justification, analytical depth, and writing clarity are needed before it is suitable for publication.

**********

what does this mean?). If published, this will include your full peer review and any attached files.

**Do you want your identity to be public for this peer review?**  If you choose “no”, your identity will remain anonymous but your review may still be made public.

For information about this choice, including consent withdrawal, please see our Privacy Policy

Reviewer #1: No

Reviewer #2: No

**********

[NOTE: If reviewer comments were submitted as an attachment file, they will be attached to this email and accessible via the submission site. Please log into your account, locate the manuscript record, and check for the action link "View Attachments". If this link does not appear, there are no attachment files.]

**Figure Resubmissions:**

Attachment Submitted filename: Review.docx

Attachment Submitted filename: Reviewers comments_PGPH-D-25-03058.pdf

# Author comment: Author response to Decision Letter 1

**DOI:** 10.1371/journal.pgph.0007534.r003

22 May 2026

Attachment Submitted filename: Response to Reviewers.docx

# Peer review: Decision Letter 1

**DOI:** 10.1371/journal.pgph.0007534.r004

**Authors:** Mahbub-Ul Alam

8 Oct 2026

Facial cleanliness, environmental factors, and the burden of trachoma in a pastoral conflict area of Tiaty East, Baringo County, Kenya

PGPH-D-25-03058R1

Dear Ms Njogu,

We are pleased to inform you that your manuscript 'Facial cleanliness, environmental factors, and the burden of trachoma in a pastoral conflict area of Tiaty East, Baringo County, Kenya' has been provisionally accepted for publication in PLOS Global Public Health.

Before your manuscript can be formally accepted you will need to complete some formatting changes, which you will receive in a follow up email. A member of our team will be in touch with a set of requests.

Please note that your manuscript will not be scheduled for publication until you have made the required changes, so a swift response is appreciated.

IMPORTANT: The editorial review process is now complete. PLOS will only permit corrections to spelling, formatting or significant scientific errors from this point onwards. Requests for major changes, or any which affect the scientific understanding of your work, will cause delays to the publication date of your manuscript.

If your institution or institutions have a press office, please notify them about your upcoming paper to help maximize its impact. If they'll be preparing press materials, please inform our press team as soon as possible -- no later than 48 hours after receiving the formal acceptance. Your manuscript will remain under strict press embargo until 2 pm Eastern Time on the date of publication. For more information, please contact globalpubhealth@plos.org.

Thank you again for supporting Open Access publishing; we are looking forward to publishing your work in PLOS Global Public Health.

Best regards,

Mahbub-Ul Alam, PhD

Academic Editor

PLOS Global Public Health

***********************************************************

The manuscript can be accepted. Please check tables and text for inconsistency. Please also check the reference for duplication.

Comments from PLOS Editorial Office: We note that Reviewer 3's comments lack relevance to the current submission. As such, we would request that the authors disregard Reviewer 3's comments.

Reviewer Comments (if any, and for reference):

Reviewer's Responses to Questions

**Comments to the Author**

Reviewer #1: All comments have been addressed

Reviewer #3: All comments have been addressed

**********

publication criteria?

Reviewer #1: Yes

Reviewer #3: Yes

**********

3. Has the statistical analysis been performed appropriately and rigorously?

Reviewer #1: Yes

Reviewer #3: (No Response)

**********

4. Have the authors made all data underlying the findings in their manuscript fully available (please refer to the Data Availability Statement at the start of the manuscript PDF file)?

The PLOS Data policy

Reviewer #1: Yes

Reviewer #3: Yes

**********

5. Is the manuscript presented in an intelligible fashion and written in standard English?

Reviewer #1: Yes

Reviewer #3: Yes

**********

**********

what does this mean?). If published, this will include your full peer review and any attached files.

**Do you want your identity to be public for this peer review?**  If you choose “no”, your identity will remain anonymous but your review may still be made public.

For information about this choice, including consent withdrawal, please see our Privacy Policy

Reviewer #1: No

Reviewer #3: **Yes:**  Gagan Matta

**********
